# Brucellosis relapse: Rate, patient characteristics, and clinical outcomes in an endemic region

**DOI:** 10.1371/journal.pntd.0013270

**Published:** 2025-07-07

**Authors:** Ibrahim Bahabri, Anadel Hakeem, Mohammed S. Alturki, Mutaz Althobaiti, Sameera Aljohani, Ahmad Alharbi, Mohammad Bosaeed

**Affiliations:** 1 Department of Medicine, King Abdulaziz Medical City, Riyadh, Saudi Arabia; 2 King Abdullah International Medical Research Center, Riyadh, Saudi Arabia; 3 Department of Cardiac Science, King Abdulaziz Medical City, Riyadh, Saudi Arabia; 4 Department of Pathology & Laboratory Medicine, King Abdulaziz Medical City, Riyadh, Saudi Arabia; 5 College of Medicine, King Saud bin Abdulaziz University for Health Sciences, Riyadh, Saudi Arabia; Yale University School of Medicine, UNITED STATES OF AMERICA

## Abstract

**Introduction:**

Brucellosis is the most common zoonotic disease worldwide, with significant heterogeneity in the literature regarding the risk factors and rate of relapse, we reviewed cases of confirmed brucellosis in Saudi Arabia, looking at the rate of relapse, characteristics of relapse cases, and compared it to the existing literature.

**Methods:**

A retrospective analysis was conducted on confirmed cases of brucellosis. Cases with suspected relapse, as noted in medical charts, were identified. A detailed review of clinical and laboratory findings, along with associated outcomes, was performed to confirm instances of brucellosis relapse. Descriptive analysis was then undertaken to characterize confirmed relapse cases.

**Results:**

Of the 1,049 confirmed brucellosis cases over eight years, 73 cases were initially suspected of potential relapse. After excluding misdiagnosis and repeat exposures cases, there were 37 cases confirmed as relapses, resulting in a relapse rate of 3.53%. When considering only cases where patients adhered to the full treatment regimen, 10 patients were considered as “true relapse” cases, reducing the relapse rate to 0.95%. Among the 10 true relapse cases, four initially presented with bacteremia, and all ten patients had elevated *Brucella* titers. None had focal infection initially. All true relapse patients received Doxycycline as the core agent, with rifampin or streptomycin as a second agent. Upon relapse, four patients developed spondylitis, and three had bacteremia. Eight patients received an extended duration and/or triple therapy, and only one patient relapsed for a third time with an infected cardiac device. No mortalities were recorded within 90 days of completing relapse treatment.

**Conclusion:**

The relapse rate observed in this study is lower than previously reported in the literature. Further research on brucellosis relapse should prioritize investigating treatment adherence and establishing a standardized definition, which might help address the current discrepancies in reported data.

## Author summary

Brucellosis is an infection caused by different species of the *Brucella* bacteria. It is transmitted from animals to humans by direct contact or by consumption of contaminated animal products. Brucellosis requires prolonged treatment with multiple antibiotics to be fully eradicated, otherwise it can relapse. How often brucellosis relapses is a topic of contention, with a wide variety of estimates. This is likely due to the lack of a standard definition of relapse, as well as the smaller sample size of some of the studies. We aimed to get an accurate estimate of the rate of brucellosis recurrence, utilizing a larger sample size of 1,049 confirmed brucellosis cases in our hospital, which is a referral center in an endemic area. We also implemented strict criteria, ensuring that other causes of therapy failure, such as nonadherence or repeated exposure, are not mislabeled as relapse. As a result, we found that the relapse rate of brucellosis was 0.95%, considerably lower than the rate in most reported studies. This demonstrates that the rate of brucellosis relapse when standard treatment regimens are adhered to are less than previously estimated.

## Introduction

Human brucellosis is an infectious disease caused by several species of the bacterial genus *Brucella*. *Brucella* is a Gram-negative intracellular organism. Brucellosis is a zoonotic disease transmitted from animals, including sheep, goat, camels and pigs, to humans by direct contact and inoculation into abrasions in the human skin and mucosa, or by consumption of contaminated unpasteurized milk and other dairy products such as fresh cheese [1]. Although not very common, other routes of transmission such as organ/tissue transplantation, blood transfusions, and sexual intercourse have been reported [2–4]. *Brucella* species causing human infection include B. melitensis, B. abortus, and B. suis [5]. A review and statistical analysis of international public health data published in 2023 gave a conservative estimate of an annual global incidence of 2.1 million cases of human brucellosis [6]. Human brucellosis is endemic in Saudi Arabia, with 37,477 total cases reported from 2004 to 2012, and an annual incidence rate ranging from 12.5 to 22.9 cases per 100,000 population annually during that period [7]. The diagnostic work-up for brucellosis remains a challenge for physicians around the globe [8]. The challenge lies in the variable clinical picture since brucellosis can manifest in many ways, mimic other diseases, and involve multiple organs, tissues, or systems [9]. The clinical course of brucellosis can be subclinical, acute, sub-acute, or chronic. Additionally, brucellosis carries a risk of relapse even after treatment [10]. Relapse is defined in the literature as the re-emergence of disease with characteristic signs and symptoms or a new positive culture 12 months after completion of the treatment regimen [11]. Several studies proposed that genetic polymorphism in cytokines regulatory regions may influence immune response levels, downregulating the immune response, and increasing the susceptibility to new infections and relapse [12,13]. Others suggest that intracellular pathogenicity of *Brucella* in which the bacterium is engulfed by macrophages plays a role [11,14]. However, the specific cause of relapse in brucellosis patients remains unknown [15]. Therefore, the identification of risk factors can generate questions that guide researchers to identify the mechanism by which relapse occurs. Studies in Spain suggested key risk factors for disease relapse, including delayed therapy initiation (symptoms lasting fewer than 10 days before treatment), positive blood cultures at baseline, and elevated temperatures (>38.3°C). These findings were consistent across two studies involving 530 and 200 adult patients [10,13].

Relapse rates in brucellosis vary significantly by region and study design. For example, looking at data from Iran A prospective trial over 2 years of 93 patients in Ahvaz city in Khuzestan Province reported a relapse rate of 18.3%; but a larger review in the Hamadan Province of 7318 cases, had a much lower rate of relapse at 6.45%; this was similar to another review of 410 cases in Qom Province with a relapse rate of 6.6% [16–18]. One of the larger reviews outside was a 10-year retrospective review of 1028 patients in Turkey, which reported a relapse rate of 4.7% overall [19]. Another review of 165 cases of brucellosis in Jordan found that 35.2% of cases had relapse [20]. Looking at local data of adults in Saudi Arabia, a study of 159 patients Hafr Al-Batin reported a relapse rate of 11.3% [21], while a cohort of brucellosis cases in Altaif had a significantly higher relapse rate of 16.5% [22]. A more recent study in Saudi Arabia, reviewing 153 pediatric patients, showed a relapse rate of 4.6% [23]. This study seeks to address the significant discrepancy between international and local data on brucellosis relapse by examining relapse rates, clinical characteristics, complications, and outcomes in patients from an endemic region in Saudi Arabia.

## Materials and methods

### Ethics statement

This study was approved by the Institutional Review Board (IRB) in the Ministry of National Guard Health Affairs under Project N. (NRC22R/368/08) at King Abdullah International Medical Research Center.

Informed consent was not required as our study is a chart review. Subjects’ privacy and confidentiality were assured, all data was kept in a secure place within the Ministry of National Guard’s premises, both hard and soft copies. No contact with patients occurred during the study period, and no additional tissue samples or blood tests were done for this study, and no personal identifiers will be published.

### Study design and setting

This was a retrospective chart review conducted in King Abdulaziz Medical City in Riyadh (KAMC-RD), a tertiary care center in Riyadh, Saudi Arabia, with over 1500 operational beds. KAMC-RD serves a diverse patient population and provides specialized care for a wide range of infectious diseases, including endemic zoonotic infections such as brucellosis. For patients with suspected brucellosis, blood or tissue samples were cultured on specialized media to isolate *Brucella* species. The isolated colonies were then analyzed using matrix-assisted laser desorption/ionization-time of flight (MALDI-TOF) mass spectrometry. Susceptibility testing of the isolates was performed using Broth microdilution or gradient diffusion (ETEST by bioMérieux). Serum agglutination test (SAT) was performed to detect antibodies against Brucella species both at the initial diagnosis and upon suspected relapse, specifically *Brucella abortus* and *Brucella melitensis*. SAT is a widely used quantitative test that provides insight into the patient’s immune response to Brucella infection, with titers ≥1:160 considered indicative of active infection in endemic settings [24].

### Patient and public involvement

Patients or the public were not involved in the design, or conduct, or reporting, or dissemination plans of our research

### Patient population

All patients 18 years or older, who were diagnosed with brucellosis between January 2015 and December 2022, were identified and screened for inclusion. Brucellosis was confirmed in these patients either by serological testing in patients exhibiting clinical symptoms compatible with brucellosis, or by positive blood culture results, consistent with the diagnostic criteria established in the literature [25].

### Definition of relapse

Relapse in this study was defined as the reappearance of characteristic signs and symptoms of brucellosis, or a new positive culture 12 months following the completion of an initial treatment regimen. This definition aligns with standard clinical guidelines and previous literature [11].

### Treatment

Standard treatment regimens were based on the 2006 WHO guidelines [26]

### Inclusion and exclusion criteria

To accurately identify cases of true relapse and reduce potential diagnostic ambiguity, only cases meeting our cited definition of relapse were included, with several exclusion criteria applied:

1. Patients with documented re-exposure to high-risk sources were excluded from being counted as “true relapse”, and instead were counted as “repeated exposure”, this included those who had been exposed again to animals known to carry brucellosis, such as camels and sheep, or who consumed unpasteurized animal products during the follow-up period.
2. Patients exhibiting signs of persistent infection, defined as continuous symptoms without a period of remission, were also excluded, and labeled as “misdiagnosis”
3. Patients in whom the diagnosis of relapse was questionable, due to concurrent febrile illnesses that might mimic or obscure the clinical signs of brucellosis relapse, alternative diagnosis, or vague clinical symptoms with no clear response to treatment were excluded from being counted as “true relapse”, and instead were counted as “misdiagnosis”.
4. Patients who were lost to follow-up within the 12-month post-treatment period were excluded.
5. Patients who did not complete the full standard treatment regimen, due to factors such as non-compliance, interruption, or early cessation of therapy, were labelled as cases of “partial treatment.” This subgroup was analyzed separately to determine whether incomplete treatment could have contributed to the relapse.

### Outcome measure

The primary outcome of interest was the relapse rate of brucellosis, calculated as the percentage of patients who met the criteria for relapse out of the total cohort of confirmed brucellosis cases identified during the study period.

### Data collection and statistical analysis

For patients meeting the criteria for brucellosis relapse, detailed data were extracted from the hospital’s electronic information system (BESTCare 2.0). Collected variables included demographic information (age, gender, occupation), clinical presentation, laboratory and serological results, treatment regimens administered, adherence and compliance with the treatment regimen, patient outcomes, and complications observed. Descriptive statistics were used to summarize the demographic and clinical characteristics of the study population. The relapse rate was expressed as a percentage.

## Results

### Rate of relapse

**Fig 1:**
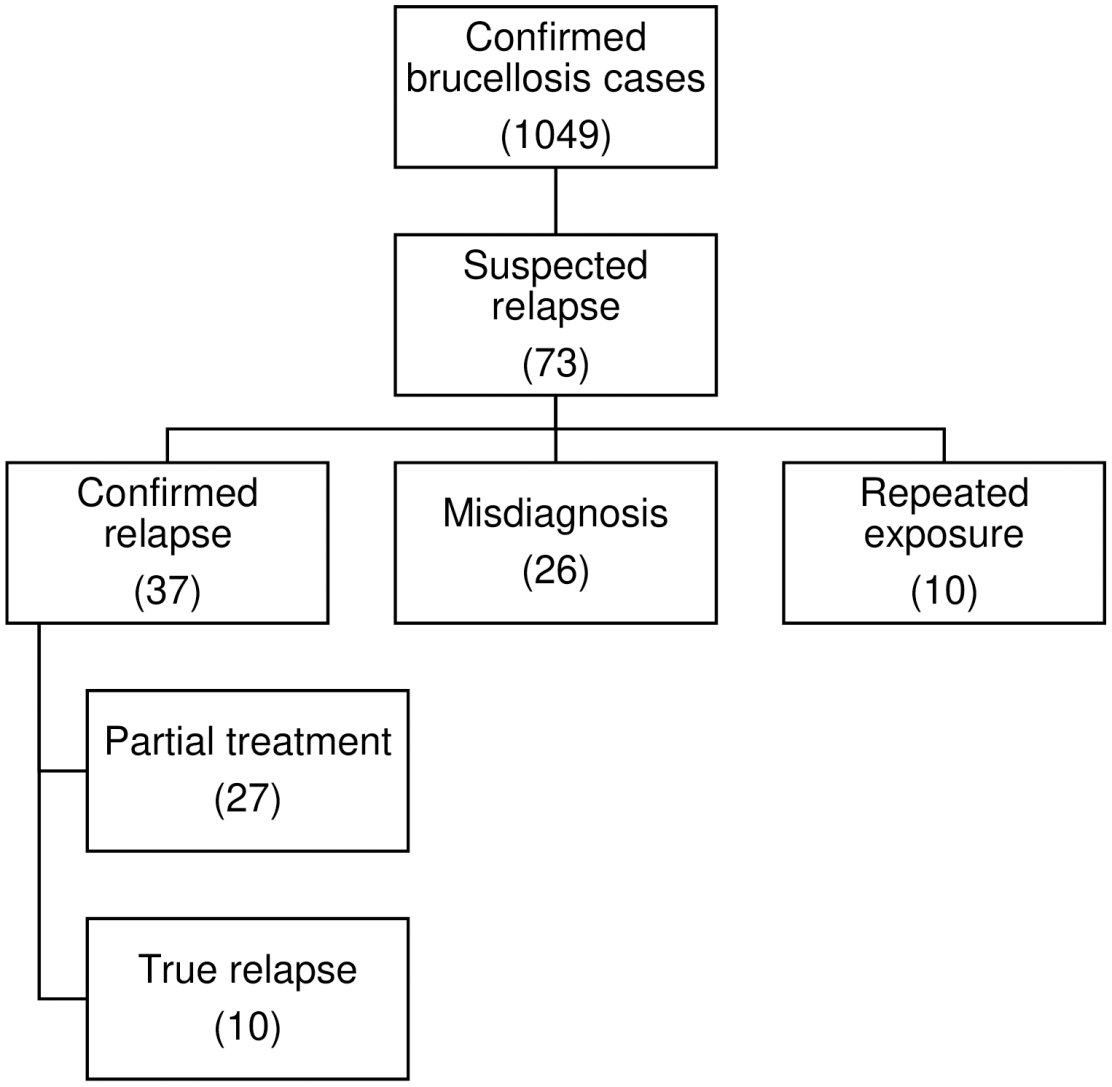
Flowchart of screening process of brucellosis cases, patients were labelled according to the study definitions based on signs and symptoms in the medical records, as well as exposures, serology and culture results.

During the eight-year study period, 1,049 confirmed cases of brucellosis were identified. Among these, 73 cases were flagged for potential relapse in the medical records. Of these, 10 cases were excluded due to repeated exposure to animal or animal products, which could lead to reinfection rather than relapse. Additionally, 26 cases were found to be misdiagnoses, where an alternative diagnosis or concurrent febrile illness was later confirmed, symptoms resolved without retreatment for brucellosis, or symptoms persisted despite receiving a second course of treatment. After excluding these cases, 37 cases met the criteria for relapse, resulting in a relapse rate of 3.53%. No patients were lost to follow-up within the 12-month post-treatment period. Details of the screening process are illustrated in Fig 1, and further details about patient classified as repeated exposure, misdiagnosis and partial treatment are in S1 Appendix.

Of the 37 relapse cases, only 10 patients had received a full standard regimen, qualifying as “true relapse” cases. When calculating the relapse rate based solely on these true relapse cases, the rate was 0.95%. The remaining 27 cases in the “partial treatment” group had either received one medication only (due to refusal of injections or intolerance to an oral agent), did not complete the full treatment duration due to an initial symptom improvement, or a combination of these factors. Seven of the partially treated patients had bacteremia upon relapse, and all isolates remained susceptible to streptomycin and tetracycline, despite all patients having received either doxycycline or streptomycin during their initial treatment. Notably, no mortalities were recorded within 90 days post-relapse for both true relapse and partial treatment group.

### Characteristics of “True relapse” cases - first brucellosis infectious episode

The 10 true relapse cases had a mean age was 56.1 years. Eight out of the 10 cases were males. One patient had heart failure with a reduced ejection fraction (HFrEF) and was on an implantable cardioverter-defibrillator (ICD), five patients had hypertension, and three had type 2 diabetes. No other significant comorbidities were noted among these patients. Six out of the 10 patients reported a history of animal contact or raw animal product consumption. All 10 patients had a reported history of fever, but only 5 had a recorded temperature reading of 38 or higher at the time of diagnosis. The average duration of symptoms before the initial diagnosis of approximately 66 days, and 7 patients had symptoms for 2 months or longer prior to starting treatment. None of the patients had focality on their initial diagnosis. Four patients had bacteremia on their first presentation, with all isolates susceptible to tetracycline, streptomycin, rifampin, ciprofloxacin and co-trimoxazole, except for one isolate that was resistant to ciprofloxacin. Titers of serum agglutination test (SAT) for *Brucella abortus* and/or *Brucella melitensis* were 1:1320 or higher for all 10 at the time of diagnosis. All 10 patients received doxycycline for six weeks as a core agent, and as a second agent, five patients were treated with streptomycin for 14 –21 days, while the other five received rifampin for six weeks. Details regarding the first episode for true relapse cases, as well as their treatment regimens, are presented in Table 1.

**Table 1:** Details of patients for the first episode of brucellosis infection.

	Age	Gender	Comorbidity	Exposure	Duration of symptoms	Temperature Celsius (°C)	Positive blood culture	Brucella titer^1^ (abortus, melitensis)	Treatment^2^
Patient 1	31	Female	None	None	2 months	37.3°	No	1:640, 1:320	Doxycycline + Streptomycin
Patient 2	69	Male	HTN	Camel contact + camel milk	2 months	37°	No	1:320, 1:320	Doxycycline + Streptomycin
Patient 3	64	Male	HTN	Sheep	1 month	36.9°	No	1:320, 1:1280	Doxycycline + Rifampin
Patient 4	65	Male	DM	None	2 months	39.4°	No	1:5120, 1:2560	Doxycycline + Rifampin
Patient 5	63	Male	DM, HTN, HFrEF on ICD	Camel milk	4 months	36.8°	Yes	1:10240, 1:10240	Doxycycline + Streptomycin
Patient 6	68	Male	HTN	None	1 month	38.7°	Yes	1:1280, 1:10240	Doxycycline + Rifampin
Patient 7	31	Female	None	Sheep + sheep dairy product	2 months	39°	No	>1;20480, >1;20480	Doxycycline + Streptomycin
Patient 8	48	Male	None	Camel milk	1 month	38.3°	Yes	1:1280, 1:640	Doxycycline + Rifampin
Patient 9	48	Male	None	Camels	4 months	37.4°	Yes	1:640, 1:1280	Doxycycline + Rifampin
Patient 10	74	Male	DM, HTN	None	3 months	38°	No	1:2560, 1:1280	Doxycycline + Streptomycin

DM, Diabetes Mellitus; HTN, Hypertension; HFrEF, Heart failure with reduced ejection fraction; ICD, implantable cardioverter-defibrillator

^1^*Brucella* serology for both species by serum agglutination test

^2^Treatment route and duration: Doxycycline oral for 6 weeks, streptomycin intramuscular for 14–21 days, rifampin oral for 6 weeks

### Characteristics of “True relapse” cases – upon relapse

The mean time to relapse diagnosis after completion of treatment was 130.2 days. Four patients developed a new focal infection upon recurrence, all manifested as spondylitis. Three patients had bacteremia upon their relapse episode. Two of the bacteremic patients also had bacteremia on their first episode. All blood culture isolates on relapse remained susceptible to the same agents tested, except for one patient, whose isolate developed streptomycin resistance following a previous course of streptomycin treatment. Six out of the 10 patients had SAT titers that were higher than the initial episode titers for at least one *Brucella* species upon relapse, one had equal titers for both species, and 3 had equal or lower titers for both species.

For almost all relapse patients, retreatment included doxycycline as a core agent in both episodes, except for one patient, who relapsed during pregnancy, and was treated with rifampin and co-trimoxazole instead. Eight patients received either triple therapy or an extended duration of two agents, four of the patients that received triple therapy or an extended duration had no new identified focality or complications. Fortunately, no mortalities were recorded within 90 days following the completion of treatment for relapse, and no further relapse episodes were recorded within 12 months of the first relapse, except for one patient, who had a third episode complicated by an endovascular infection. Further information on the relapse episodes, complications and treatment regimens, is provided in Table 2, and detailed data for the true relapse as well as partial treatment cases can be found in S2 Appendix.

**Table 2:** Details of patients’ relapses, patients are listed in the same number and order as in Table 1.

	Time to relapse (days)^1^	Focality on relapse	Temperature Celsius (°C)	Positive blood culture	Brucella titer^2^ (abortus, melitensis)	Treatment
Patient 1^*^	154	None	37.1	None	1:640, 1:320	Rifampin 70 days + co-trimoxazole 70 days
Patient 2	77	None	37	None	1:640, 1:640	Doxycycline 90 days + ciprofloxacin/rifampin 90 days^#^ + streptomycin 14 days
Patient 3^**^	189	Spondylitis with abscesses	37.1	None	1:2560, 1:10240	Doxycycline 210 days + rifampin 210 days + streptomycin 14 days
Patient 4	139	None	37.2	None	1:320, 1:160	Doxycycline 42 days + streptomycin 21 days
Patient 5^+^	281	Spondylitis	37.4	Yes	1:5120, 1:20480	Doxycycline 120 days + co-trimoxazole 120 days
Patient 6	182	Spondylitis	37.2	None	1:2560, 1:5120	Doxycycline 180 days + rifampin 180 days + streptomycin 10 days
Patient 7	96	None	38.3	Yes	1:1280, 1:640	Doxycycline 42 days + rifampin 42 days +streptomycin 14 days
Patient 8	38	None	38.5	None	1:2560, 1:5120	Doxycycline 42 days + streptomycin 14 days
Patient 9	51	None	37.7	Yes	1:320, 1:320	Doxycycline 60 days + streptomycin 21 days
Patient 10	95	Spondylitis	36.9	None	1:2560, 1:5120	Doxycycline 90 days + rifampin 90 days + streptomycin 21 days

^1^From completion of treatment until diagnosis of relapse

^2^*Brucella* serology for both species by serum agglutination test

* Relapsed during pregnancy

** Patient had non-drainable bilateral psoas abscesses in continuation with an epidural abscess, completely resolved on follow up imaging at the end of therapy with no other interventions

+ Relapsed for a 3^rd^ time with an infected cardiac device 71 days after his second episode, his third episode was treated with device removal then doxycycline, rifampin and ciprofloxacin for 8 months post-surgery

# Ciprofloxacin was given for 30 days, then switched to rifampin for 60 more days due to GI intolerance

## Discussion

The rate of true brucellosis relapse in our study of 0.95% was lower than most reports in the literature. It remains lower even with the inclusion of partially treated patients, which brings up the relapse rate to 3.53%. The significant variations are likely due to the high differences in the sample size, study design and setting, and the case definitions. Of note, the largest trial in adults with a similar design and sample size to ours, by Buzgan et al., had a closer relapse rate to ours at 4.7% than most other reports in the literature, and the two of the other larger studies in Iran, had low relapse rates of 6.45-6.6% [17–19]. In contrast, significantly higher relapse rates, ranging widely from 11.3% to 35.2%, were typically observed in studies with smaller sample sizes [16,20–22].

There are additional challenges when attempting to compare data across studies, such as the absence of a standardized definition in the literature. For instance, one study defined all relapse cases by having a rise in *Brucella* titer along with recurrence of their symptoms. However, we know from our data that this is not always the case; as we had three patients with lower titers on relapse, and diagnoses were confirmed in 2 of them through positive blood cultures at the time of relapse [20]. Another issue is the focus on different variables across studies. For example, in most larger studies, including those relapse rates similar to ours, there was no clear commentary on whether adherence was assessed. This could lead to cases that did not receive full treatment being counted as relapse; while we demonstrated in our study that many cases labelled as relapse had only received partial therapy [17–19]. Furthermore, it is well documented in the literature that treatment with monotherapy or for short durations is associated with a higher treatment failure [27,28].

Looking at general characteristics of our patients, 8 out of the 10 patients in our relapse group were males. This aligns with findings from other studies where the majority of relapse cases were males, suggesting a potential male predominance in relapse cases [16–18]. However, it is important to mention that local and international data regarding brucellosis in general shows a male predominance, which could account for males being overrepresented in the relapse group, rather than male gender being a risk factor for brucellosis relapse [29–32]. None of the relapse patients had osteoarticular disease or other established focality on their initial episode, which is an established risk factor for relapse [19]. One reason that no patients with initial focality were identified in our true relapse cases, was the fact that these patients had partial treatment due to difficulty completing the standard prolonged regimens of three medications over 12 weeks, and so in our study they were classified as “partial treatment”, where they would have been counted as relapsed cases in other studies.

Only 4 out the 10 relapsed cases had bacteremia on their initial episode, and only 3 had bacteremia on relapse, much lower rates of bacteremia initially and on relapse, compared to a 16-year prospective study on brucellosis relapse cases, during which ∼80% of cases had documented bacteremia during their initial disease, 65% had bacteremia on relapse, and positive blood cultures on initial disease were suggested to be an independent risk factor for relapse, with an odds ratio (OR) of 2.6 [33]. All patients in our true relapse group had a reported history of fever, which is an established risk factor for relapse from previous data [18]. When it comes to the grade of recorded temperature, only 5 patients in our study had a documented fever of 38.0 or higher, and only 3 out of the 5 patients with recorded fevers had a reading of 38.3 or higher, which was established as a cutoff for a predictor of relapse previously [14]. An intriguing variable for predicting relapse risk is the duration of the presenting symptoms. In our study, the mean duration from the symptom’s onset to the initiation of treatment was 66 days, with 7 out of the 10 patients experiencing symptoms for two months or longer. This contrasts with a prospective study where a ⩽ 10-day duration of the disease before treatment was an independent risk factor for relapse with an odds ratio of 1.9. However, our findings align with two other studies, one showed that delayed treatment (>50 days) increasing relapse rate more than fourfold based on multiple logistic regression analysis, and another where the 70.6% of relapse cases had symptoms lasting longer than three months prior to diagnosis [11,16,18].

One concern when treating bacterial infections is the development of resistance to previously used antibiotics. Most isolates in our study were susceptible to all tested antimicrobials, including agents previously used by the patients. The only exceptions were an isolate resistant to ciprofloxacin, and another resistant to streptomycin in a patient who had previously received streptomycin. For comparison, a recent brucellosis data in Middle Eastern and North African (MENA) countries showed that most human isolates remained susceptible to doxycycline and aminoglycosides. However, it also highlighted growing resistance to rifampin, decreased susceptibility to co-trimoxazole, and variable activity of ciprofloxacin due to inconsistent reported minimum inhibitory concentrations (MICs). Furthermore, therapeutic failures with fluoroquinolones were noted when used for treatment of brucellosis, supporting the use of a tetracycline-aminoglycoside-based regimen when susceptibility is uncertain [34].

Regarding treatment regimens, all patients in our study received doxycycline. For the second agent, the 10 patients were evenly split between streptomycin and rifampin. This is aligned with previous data of none of the standard regimens being shown to have a significantly higher relapse rate when used [18].

The key strengths of our study include the large sample size of brucellosis cases, and the retrospective chart review design, which benefited from the use of advanced electronic medical records (BESTCare 2.0). This allowed for detailed and accurate assessment of the clinical information including treatment adherence and diagnosis confirmation, ensuring that cases of relapse were true relapse cases, rather than undertreatment or misdiagnoses. This is evident from the initial identification of 73 suspected relapse cases, which was refined to a final count of true relapse cases after rigorous scrutiny using the electronic medical records (EMR) system. The main limitation of our study is the small number of true relapse cases. This is why we were unable to perform a robust statistical analysis, to establish statistically significant risk factors for relapse. Another issue is the retrospective nature, with missing data from previous physician documentation about issues such as exposures, and a full review of systems, as well as certain investigations (for example, blood cultures), not being performed for all patients. Nonetheless, characteristics of patients’ initial disease, and treatment regimens in our study align with previously published data on relapse cases as discussed. The retrospective nature of our study also meant that we could not perform further testing that could provide useful data and insights in confirming relapse, such as detection of *Brucella* Deoxyribonucleic Acid (DNA) by polymerase Chain Reaction (PCR), or repeating SAT titers at 2-week intervals to detect a fourfold difference for antibody titres between acute and convalescent-phase serum. Although we did attempt to mitigate this limitation with strict inclusion/exclusion criteria where we only counted patients that had compatible signs and symptoms at the time of relapse, with no other identified cause of their symptoms, and a clear response to retreatment, this is still an important limitation to keep in mind.

One area for future research is the establishment of the national registry of complicated and relapsed cases in our country, similar to initiatives done in other regions with higher brucellosis endemicity [35]. Such a registry would help ensure the adoption of a unified standardized definition of relapse, and with the larger cumulative sample size over time, enable more accurate characterization of brucellosis relapse cases. Additionally, it would facilitate better identification and validation of risk factors for relapse.

## Conclusion

The overall rate of brucellosis relapse cases in our study was significantly lower than previously reported. This finding may be attributed to overestimation of relapse cases, where some important factors like medication adherence and misdiagnosis were not ensured. Completing the recommended longer duration combination therapies for brucellosis is essential to minimize the risk of relapse. Notably, there were no recorded mortalities among relapse cases, and all patients achieved complete cure without further relapse once therapy was extended and infection foci were addressed. Additional research is needed to determine the true rate of brucellosis relapse and to identify its associated risk factors.

## Supporting information

S1 Appendix Brief description of suspected relapse cases and their category (true relapse, partial treatment, re-exposure, misdiagnosis), medical record number removed to preserve patient anonymity. (XLSX)

S2 Appendix Patient Data of true relapse cases and partial treatment cases, medical record number removed to preserve patient anonymity. (XLSX)
